# LCN2 is a new diagnostic biomarker and potential therapeutic target in idiopathic short stature

**DOI:** 10.1111/jcmm.17408

**Published:** 2022-05-24

**Authors:** Xijuan Liu, Jian Zhang, Jinghong Yuan, Rui Ding, Tao Liu, Jingyu Jia

**Affiliations:** ^1^ Department of Pediatrics The Second Affiliated Hospital of Nanchang University Nanchang China; ^2^ Department of Orthopedics The Second Affiliated Hospital of Nanchang University Nanchang China; ^3^ Department of Pediatrics The First Affiliated Hospital of Nanchang University Nanchang China

**Keywords:** biomarker, endochondral ossification, growth plate, idiopathic short stature, Lipocalin 2

## Abstract

Idiopathic short stature (ISS) is the most common paediatric endocrine disease. However, the underlying pathology of ISS remains unclear. Currently, there are no effective diagnostic markers or therapeutic strategies available for ISS. In this study, we aimed to identify differential plasma protein expression and novel biomarkers in patients with ISS, and elucidate the biological functions of candidate proteins in ISS pathogenesis. Four specimen pairs from four ISS children and age‐/sex‐matched control individuals were subjected to proteomics analysis, and 340 samples of children with a mean age 9.73 ± 0.24 years were utilized to further verify the differentially expressed proteins by enzyme‐linked immunosorbent assay (ELISA). The receiver‐operating characteristic (ROC) curve and the area under the ROC curve (AUC) were plotted. A total of 2040 proteins were identified, of which 84 were differentially expressed. In vitro and in vivo experiments confirmed the biological functions of these candidate proteins. LCN2 overexpression in ISS was verified using ELISA. Meanwhile, LCN2 showed high sensitivity and specificity in discriminating children with ISS from those with growth hormone deficiency, precocious puberty and normal control individuals. The upregulated expression of LCN2 not only suppressed food intake but also impaired chondrocyte proliferation and bone growth in chondrocytes and rats. As a result, the rats presented a short‐stature phenotype. Subsequently, we found that bone growth inhibition recovered after LCN2 overexpression was stopped in immature rats. To our knowledge, this is the first study to report that LCN2 may be a significant target for ISS diagnosis and treatment.

## 1 INTRODUCTION

Idiopathic short stature (ISS) is the most common disease in paediatric endocrine clinics. It is defined by a standing height that is less than two standard deviations (SDs) from the corresponding mean height for age, sex and population with a normal body size at birth and without evidence of a systemic disease, nutritional, psychological, or chromosomal disorder, or overt hormonal abnormalities.^1^, ^2^ The underlying pathology of ISS is still unclear.^1^, ^2^, ^3^ Although recombinant human growth hormone (rhGH) has been used to treat ISS for many years, its treatment effects vary considerably among studies.^4^, ^5^, ^6^, ^7^

Unlike short stature associated with growth hormone (GH) deficiency, ISS patients show no significant absence of GHs. The growth hormone–insulin‐like growth factor‐I (GH‐IGF‐I) axis not only regulates longitudinal bone growth but is also involved in the pathogenesis of endocrine and cardiovascular diseases and tumours. Therefore, the issue of long‐term rhGH safety has been a concern, especially in high‐dose rhGH treatment. Carel et al.^8^ reported long‐term mortality after rhGH treatment in 6928 children with ISS, idiopathic isolated GH deficiency, neurosecretory dysfunction and small‐for‐gestational age. The mortality rates of subarachnoid or intracerebral haemorrhage, cardiovascular diseases and cancers of the bone and cartilage were significantly increased in their study. Furthermore, Ying et al.^9^ identified a 21.5% chance of hyperglycemia and a 17% chance of hyperinsulinemia after rhGH treatment in ISS patients. Moreover, they^10^ found that the patients with ISS showed a significantly higher incidence of hyperinsulinemia than the patients with GH deficiency (15.33% vs. 7.84%) after rhGH treatment. In addition, Silvers et al.^11^ pointed out that over 500,000 patients with ISS are treated with rhGH at a cost of $10 billion per year in the USA. This imposes a substantial economic burden on the families of children with ISS. The unclear pathogenesis is responsible for the aforementioned findings.

Adult height is regulated by multifactorial conditions such as genetic and environmental factors.^2^, ^3^, ^12^ A series of studies have indicated that variants of the *ACAN*,^13^, ^14^ *GHR*,^15^, ^16^ *CYP26C1*,^17^ *AKNRD11*,^18^ *NPR2*,^19^ *SHOX* and *IGFALS* ^20^genes are related to ISS pathogenesis. Although these findings may play a vital role in ISS subcategorization and treatment decisions, the incidence of gene variants is sometimes relatively low. For example, the incidence of *ACAN* variants in ISS is 1.4%,^14^ that of GHR variants is 5%,^15^ and that of *NPR2* variants is 6%.^19^ Currently, ISS diagnosis can only be defined by excluding other recognized diseases owing to the lack of specific positive findings and effective biomarkers. To improve the curative effect and long‐term safety of the rhGH treatment and increase the diagnostic sensitivity and specificity of ISS, the pathogenesis of ISS should be further investigated. Blood contains multifactorial factors that regulate long bone growth. Furthermore, plasma proteomics have been confirmed to play a vital role in revealing the pathogenesis of diseases and identifying new drug targets and candidate proteins for biomarkers.^1^, ^20^, ^21^, ^22^ However, to date, the plasma proteomics of ISS have not been adequately addressed.

The purpose of the present study was to determine the differential protein expression profile of patients with ISS, identify novel biomarkers and verify the biological function of candidate proteins in ISS pathogenesis via in vivo and in vitro experiments.

## 2 METHODS AND MATERIALS

### 2.1 Participant characteristics and plasma samples

Samples of discarded blood from routine blood tests in the ISS, precocious puberty, hormone deficiency, pneumonia and tracheitis were collected from October 2018 to March 2020. Blood specimens were centrifuged and kept at −80°C until further use. The parents/guardians of the children were informed that the collected blood samples would be used for scientific research, and written informed consent was obtained from them. Three hundred and forty children, including 68 ISS, 68 precocious puberty, 68 growth hormone deficiency, 68 pneumonia and tracheitis and 68 age‐/sex‐matched control individuals with a mean age of 9.73 ± 0.24 years, were randomly selected from our databases (Table 1). Detailed information on the demographic and clinical parameters is presented in Table 1. Four specimen pairs from four children with ISS and age‐/sex‐matched control individuals were subjected to proteomic analysis, and all 340 samples were used to verify the differentially expressed proteins by enzyme‐linked immunosorbent assay (ELISA). The differentially expressed proteins, LCN2 and CRP, were further verified in additional independent 40 samples using the human XL Cytokine Proteome Profiler™ Array Kit (ARY022b, R&D Systems) and ELISA. The biological replicates were performed in 20 ISS and 20 normal control children with a mean age of 9.09 ± 0.36 years. To analyse the diagnostic efficiency of LCN2 in discriminating ISS children from short stature caused by other common reasons, such as growth hormone deficiency and precocious puberty, the authors compared the expression of LCN2 in the ISS children, growth hormone deficiency, precocious puberty and normal control children. Several studies^23^, ^24^ have reported that bacteria inflammation can induce the overexpression of LCN2. Because the authors wanted to know whether the LCN2 upregulation in ISS was caused by bacteria infection, we compared the LCN2 expression of children with infectious diseases, such as pneumonia and bronchitis, with that of ISS children. As a result, the children of ISS, precocious puberty, hormone deficiency, pneumonia and tracheitis were involved in the present study. The ISS and GH deficient were diagnosed according to previous reports^25^, ^26^ and the guidelines of American Paediatric Endocrine Society.^27^ Two GH‐stimulation tests (Arginine and Levodopa) were performed on subsequent days in our hospital. If peak GH concentration is <10 μg/L, GH deficiency is considered according to the guidelines. If peak GH concentration is >10 μg/L, ISS is considered. The mid parental height of ISS and GH deficiency was shown in Table S2 and S3. Ethical approval for this study was obtained from the Human Research Ethics Committee of the Second Affiliated Hospital of Nanchang University, Nanchang, China. Considering that all subjects were below 12 years of age, written informed consent was obtained from their parents/guardians before enrollment.

**TABLE 1:** The information of patients and healthy individuals

Category	Males	Females	Age/m (mean ± SD)	Height/cm (mean ± SD)	Weight/Kg (mean ± SD)
ISS	31	37	9.97 ± 0.29	122.9 ± 1.72	24.72 ± 0.94
Control	36	32	9.73 ± 0.24	133.7 ± 1.28	34.94 ± 0.96
GDH	40	28	9.02 ± 0.27	123.4 ± 1.55	21.55 ± 0.62
PP	35	33	8.63 ± 0.19	130.9 ± 1.17	27.74 ± 0.59
Pneumonia	37	41	8.38 ± 0.43	133.3 ± 1.47	30.12 ± 0.63

### 2.2 Protein preparation, iTRAQ labeling and D‐LC–MS/MS

Eight specimens from age‐/sex‐matched ISS and control children were marked ISS1, ISS2, ISS3, ISS4, Con1, Con2, Con3 and Con4 and were divided into the ISS and control groups. Detailed information on protein preparation, iTRAQ labeling and D‐LC–MS/MS is provided in supplementary materials.

### 2.3 Protein screening and bioinformatics analysis

The protein screening criteria were as follows: removal of blank values, unique peptide≥1 and score Sequest HT >0. Differentially expressed proteins (DEPs) were identified according to a fold‐change (FC) ≥ 1.2 and *p*‐value <0.05. The results are shown as volcano plots. The heat map of DEPs was constructed by hierarchical clustering using the heatmap package V1.0.12 (https://cran.rproject.org/web/packages/pheatmap/index.html) in R software V3.6.0 (https://www.r‐project.org). The protein–protein interaction network was constructed using STRING (https://string‐db.org). Biological replicates of LCN2 and CRP were performed using the human XL Cytokine Proteome Profiler Array Kit (ARY022b, R&D Systems) according to the manufacturer's instructions.

### 2.4 ELISA

An ELISA kit (SED207Hu, Cloud‐Clone Corp.) was used to further verify the validity of candidate proteins in compliance with the manufacturer's protocol. The optical density of each well was measured using a microplate reader at 450 nm.

### 2.5 Culturing of chondrocytes

Human chondrocytes were supplied by the Procell Cell Resource Center (Wuhan, China). The chondrocytes were cultured in DMEM (Gibco, Thermo Fisher Scientific Inc.) containing 10% fetal bovine serum (FBS, Gibco, Australia) and maintained in a humidified incubator with 5% CO_2_ at 37°C.

### 2.6 LCN2‐overexpressing vectors and rats

The LCN2 plasmid (GV358‐Ubi‐MCS‐3FLAG‐SV40‐EGFP‐IRES‐puromycin) was successfully constructed by GENEchem Co. Ltd. (Figure S1). The chondrocytes were grown in a six‐well plate until they reached 80% confluence and then transfected with the LCN2 plasmid using Lipofectamine 3000 (Invitrogen). Empty vector‐transfected cells were used as a control group. The transfection efficiency of LCN2 was measured using fluorescence microscopy (Figure S2). The chondrocytes were collected after 72 h of transfection, and the final concentration of LCN2 was determined to be 50 nM. All assays were performed in triplicates. LCN2 was overexpressed in rats via daily intraperitoneal administration (150 ng/g) for 20 or 40 days.

### 2.7 Cell proliferation assay, EdU assay, Alkaline phosphatase and Von Kossa staining

LCN2 chondrocytes were grown in a six‐well plate until 80% confluence was reached. Proliferation rates were evaluated using the CCK‐8 assay (TransGen Biotech). The chondrocytes (2 × 10^3^) were grown in 96‐well plates and incubated in a humidified 5% CO_2_ atmosphere at 37 °C. After incubation for 24 h, the chondrocytes in each well were treated with 10 μl CCK‐8 and then incubated for another 2 h. Finally, the absorption was measured spectrophotometrically at 450 nm (VARIOSKAN FLASH, Agilent, Santa Clara, CA, USA). The chondrocytes were incubated with 5‐ethynyl‐2′‐deoxyuridine (EdU; Guangzhou Ribobio Co., Ltd., Guangzhou, China) for 2 h in compliance with the manufacturer's protocol. Subsequently, the chondrocytes were exposed to 200 μl 1xApollo reaction mixture for 25 min and then stained with Hoechst 33342 (5 μg/ml) for 30 min. Finally, the stained chondrocytes were examined by fluorescence microscopy and imaged. The alkaline phosphatase and von Kossa staining protocols used were as described in our previous study^26^ and are also given in supplementary materials.

### 2.8 RT‐qPCR and Immunoblotting

An Eastep Super RNA Extraction Kit (Promega Biotech Co., Ltd.) was used to extract total RNA from each frozen specimen. Primers used are listed in Table S1. Total protein was isolated from the chondrocytes of the two groups via cell lysis (Total Protein Extraction Kit, Applygen Technologies, Inc.) and then measured using a BCA assay (Thermo Fisher Scientific Inc.). Detailed information is provided in Supplementary Material.

### 2.9 Animal feeding and grouping

A total of 13 pregnant rats and 120 pups were included in this study. The rats were fed a mixed diet (Beijing Keao Xieli Feed Company) at 22–24°C under a 12:12‐h light/dark cycle. The flowchart (Figure S3A,B) provides detailed grouping information. Ethical approval for animal experiments was obtained from the Animal Ethics Committee of Nanchang University (Nanchang, China).

### 2.10 Histomorphological analysis, in situ hybridization, BrdU and calcein staining

Femur and tibia samples were extracted after euthanasia via an intraperitoneal injection of pentobarbital (160 mg/kg). One‐third of the distal femur and proximal tibia were resected and stored in 4% formaldehyde solution or liquid nitrogen. The specimens were decalcified with 10% EDTA for 2 months. One specimen was then cut into two equal coronal sections, which were embedded in paraffin. Serial sections of 4 μm thickness were obtained for haematoxylin–eosin and safranin O, immunohistochemistry (IHC) analysis, in situ hybridization and BrdU and calcein staining. The staining procedures used were as described in our previous study^28^ and are also given in supplementary materials.

### 2.11 Statistical analysis

The clinical parameters and differential expression of candidate genes were analysed using an unpaired *t*‐test, analysis of variance (anova) followed by the Tukey's test or chi‐squared test. To further analyse the diagnostic efficiency of LCN2 in discriminating the children in the ISS group from those in the hormone deficiency, precocious puberty and normal control groups, we conducted a receiver operating characteristic (ROC) analysis. The ROC curve and the area under the ROC curve (AUC) were calculated using MedCalc for Windows, version 19.3.0 (MedCalc Software, Ostend, Belgium). The correlation of serum LCN2 levels with age was assessed via Pearson's correlation analysis in the ISS and normal control groups. *p*‐Values of <0.001 (***), 0.01 (**) and 0.05 (*) were deemed statistically significant. All statistical tests were performed using the SPSS V20.0 software (IBM Corp.).

## 3 RESULTS

### 3.1 Proteomics analysis identified the DEPs

Proteomics analysis was conducted on four pairs of children with ISS and age‐ and sex‐matched subjects. We screened approximately 2040 proteins, of which 84 proteins were differentially expressed (60 upregulated and 24 downregulated) according to fold>1.2 and P < 0.05 between the children with ISS and control subjects (Figure S4A–C and Table S4). The top 30 Kyoto Encyclopedia for Genes and Genomes (KEGG) pathways and Gene Ontology (GO) enrichment terms are listed in Figure S4D,E, respectively. Of the 84 proteins, 40 were successfully enriched using the KEGG pathway analyses. Nineteen DEPs were enriched in the immune system with a maximum occupancy of 47.5% (19/40).

### 3.2 Plasma LCN2 is upregulated and has high diagnostic efficiency in ISS children

**FIGURE 1:**
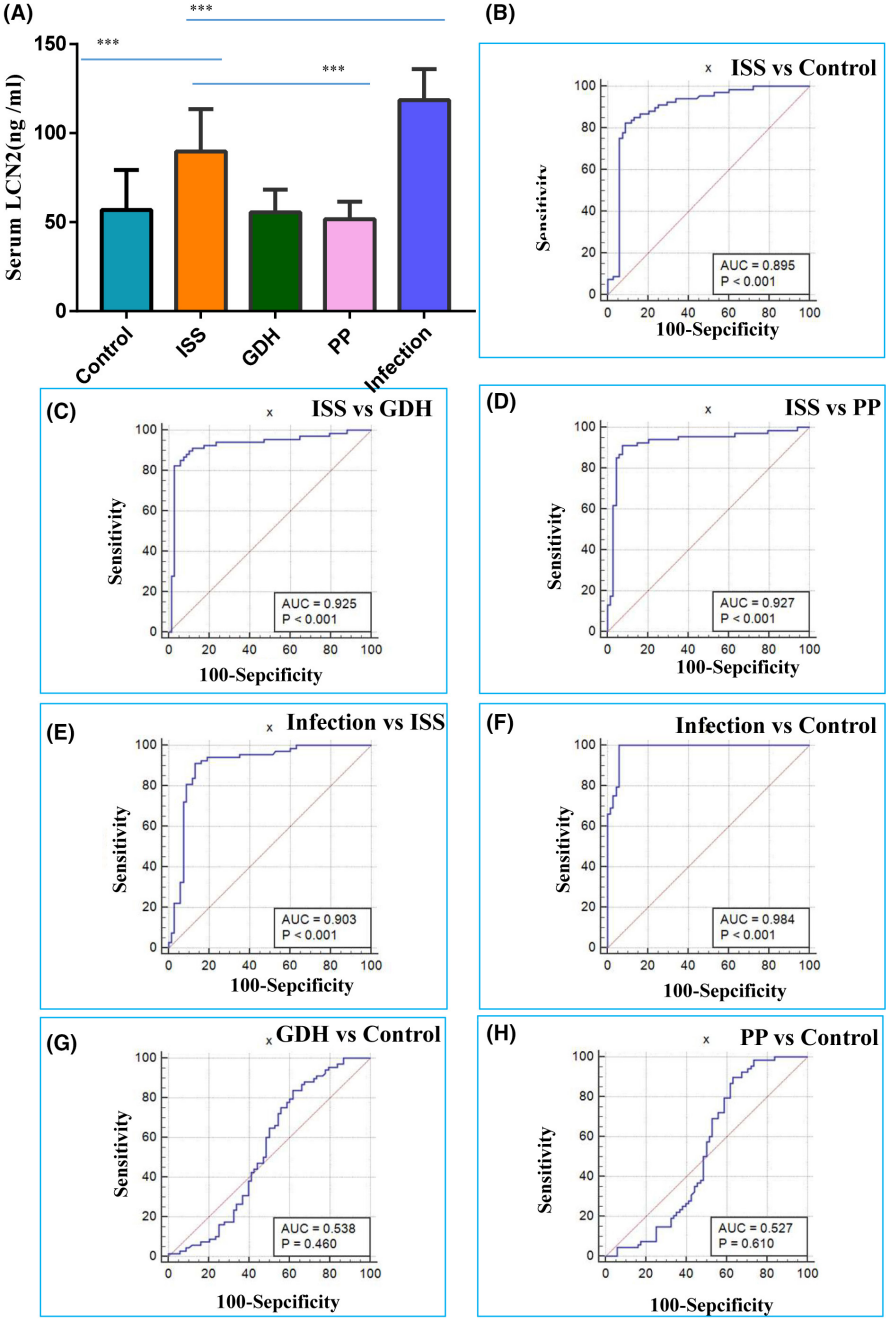
LCN2 overexpression was confirmed, and it presented a high sensitivity and specificity for the diagnostic of ISS children. (A) LCN2 overexpression was verified via ELISA in 68 ISS children and 68 normal control individuals. One‐way anova indicated LCN2 expression in ISS children was obviously higher than that in growth hormone deficiency, precocious puberty and normal participant groups, but no significant difference in LCN2 expression was observed among the hormone deficiency, precocious puberty and normal participants. This suggests that LCN2 can be considered a specific biomarker for the diagnosis of ISS. (B‐H) ROC curves and AUCs were drawn. Plasma LCN2 displayed a high diagnostic ability for discriminating ISS children from growth hormone deficiency, precocious puberty and normal control individuals. The data are presented as the mean ± SD. *n* = 3. Two groups were compared using *T*‐test, or three groups were compared using anova followed by the Tukey's test. ***p < 0.001 vs. control. AUC, area under the ROC curve; ISS, Idiopathic idiopathic short stature; LCN2, Lipocalin 2; ROC, receiver‐ operating characteristic

Given the high correlation between inflammation and immunity with ISS pathogenesis, the top 10 inflammation‐related proteins were selected to further verify the validity of the ELISA in 68 children with ISS and 68 normal control individuals. Of these, four proteins, LCN2, IGHM, C1QA and C1QB, were confirmed to be upregulated. LCN2 exhibited the most significant difference (1.63‐fold) (Figure 1A). Subsequently, one‐way anova was conducted for LCN2 between the ISS, growth hormone deficiency, precocious puberty and normal control individuals. The outcomes indicated that LCN2 expression in children with ISS (89.64 ± 2.89 ng/ml) was obviously higher than that in the GH deficiency (55.54 ± 1.54 ng/ml), precocious puberty (51.68 ± 1.18 ng/ml) and normal control (56.82 ± 2.72 ng/ml) groups. However, there was no obvious difference in LCN2 expression among the GH deficiency, precocious puberty and normal participants (*p* = 0.67). Moreover, serum LCN2 levels were not correlated with sex or age in ISS and normal control individuals (*r* = 0.099, *p* = 0.252; *t* = 1.772, *p* = 0.079). To further analyse the diagnostic efficiency of LCN2 in discriminating children with ISS from normal control individuals, ROC curves and AUCs were plotted. An AUC of 0.895 (95% CI: 0.831–0.941, *p* = 0.001) was identified in the present study. LCN2, with the best cutoff point of >88.73 ng/ml, presented a specificity of 82.35% and a sensitivity of 91.18%. In addition, plasma LCN2 has a high diagnostic ability for discriminating children with ISS from GH deficiency, precocious puberty and normal control individuals (Figure 1B–H).

**FIGURE 2:**
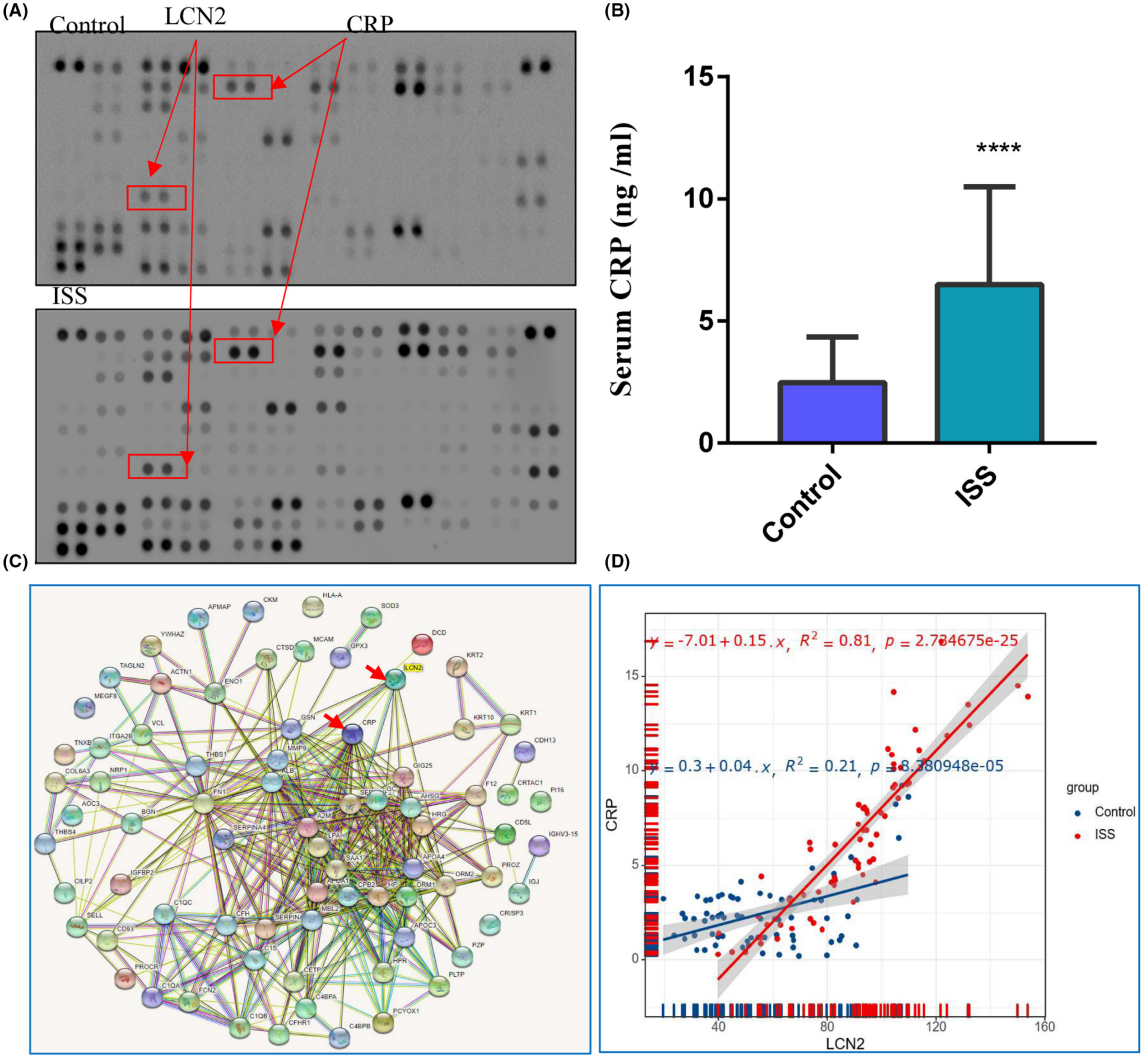
LCN2 were positively related with CRP in ISS children. (A, B) The biological replicates in additional independent samples showed that C‐reactive protein in ISS is still higher than that in normal control children (2.82‐fold and *p* < 0.0001). (C) Protein–protein interaction network was constructed using STRING databases. (D)The positive correlation between LCN2 and CRP was identified by statistical analysis. The data are presented as the mean ± SD. *n* = 3. *T*‐test, *p < 0.05, **p < 0.01, ***p < 0.001 vs. control

It has been confirmed that bacterial infectious diseases, such as pneumonia and bronchitis, can result in the overexpression of plasma LCN2. To analyse whether LCN2 is upregulated in ISS because of infectious diseases, we compared LCN2 expression between children with ISS and children with pneumonia and bronchitis. Plasma LCN2 expression in children with pneumonia and bronchitis (118.6 ± 2.09 ng/ml) was significantly higher than that in children with ISS (26.01 ± 3.06 ng/ml) (Figure 1A, *p* = 0.001). This suggests that the increased LCN2 level in children with ISS cannot be attributed to general bacterial infectious diseases. Interestingly, although routine blood tests and C‐reactive protein (CRP) levels in children with ISS were within the normal range of clinical reference values, the CRP levels in the ISS group were higher than that in the normal control group **(**2.82‐fold and *p* < 0.0001) (Figure 2A,B). Biological replicates were performed using additional independent samples to further verify the results. The LCN2 and CRP levels were still higher in the ISS group than in the normal control group. The protein–protein interaction network revealed a correlation between Lcn2 and CRP (Figure 2C). Subsequently, positive correlations were identified using a statistical analysis (Figure 2D and *p* < 0.001). No symptoms of influenza or bacterial infection, such as fever, were observed in children with ISS.

### 3.3 LCN2 suppresses proliferative chondrocytes, hypertrophic features and endochondral ossification in vitro

**FIGURE 3:**
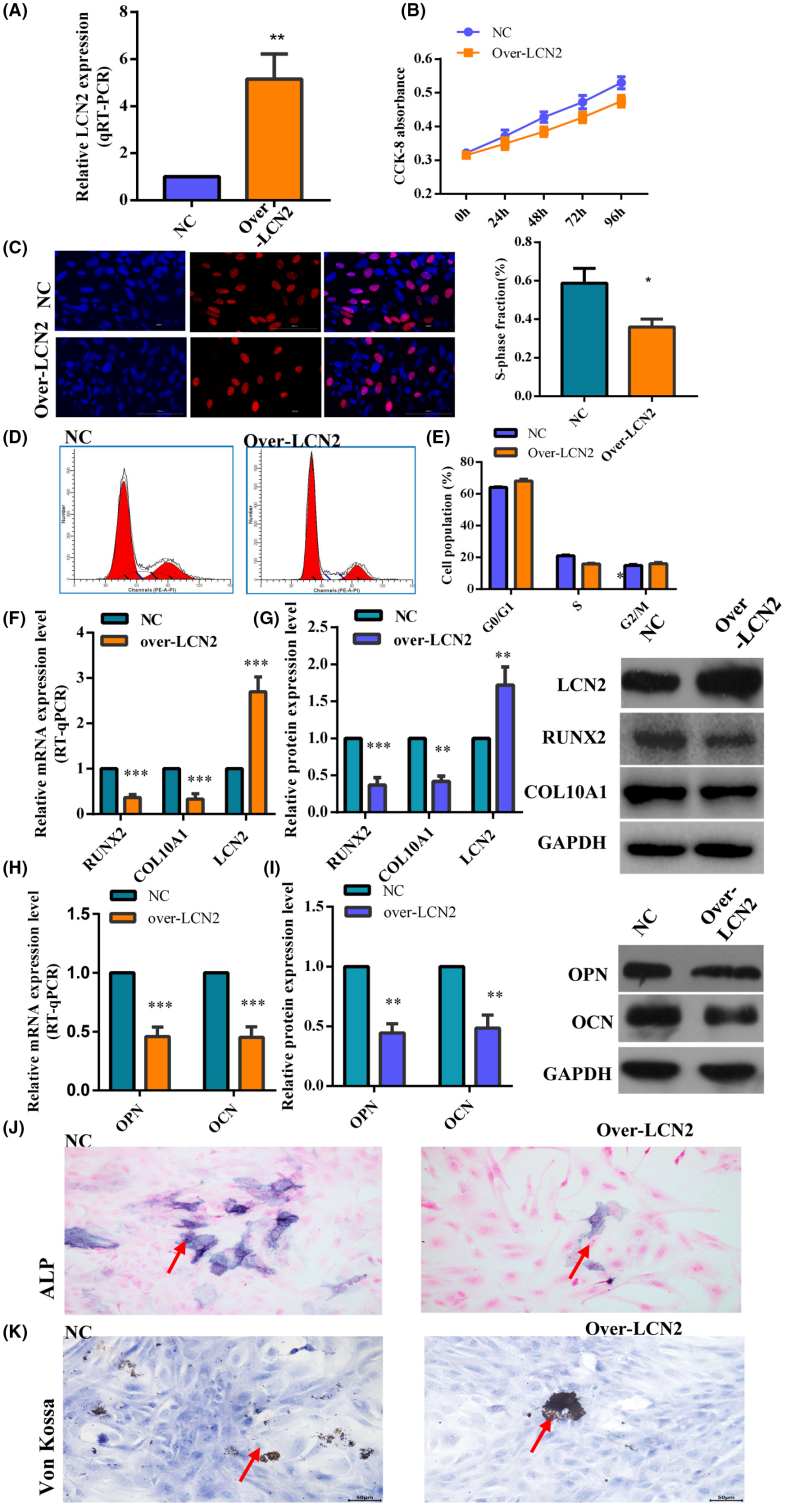
Overexpression of LCN2 inhibits proliferation and hypertrophy of chondrocyte and endochondral ossification. (A) LCN2 was overexpressed following transfection of human chondrocytes with GV358‐LCN2. (B, C) CCK‐8 and EDU assays demonstrated that overexpression of LCN2 repressed chondrocyte proliferation. (D, E) The flow cytometry assay indicates that the cell phase was arrested in the G0/G1 phase. (F–I) The expression of collagen type X, RUNX2, OCN and OPN was suppressed after LCN2 overexpression. (J, K) ALP activity was decreased, and von Kossa staining demonstrated reduced mineralization after LCN2 overexpression. The data are presented as the mean ± SD. *n* = 3. *T*‐test, *p < 0.05, **p < 0.01, ***p < 0.001 vs. control. CCK‐8, Cell Counting Kit‐8; EdU, 5–ethynyl–2′–deoxyuridine; LCN2, Lipocalin 2; OCN, osteocalcin; OPN, osteopontin; RUNX2, runt‐related transcription factor 2

To further clarify whether upregulated LCN2 is the cause or result of ISS, we upregulated the expression of LCN2 in chondrocytes. Human chondrocytes were transfected with GV358‐LCN2, and the transfection efficiency was assessed by fluorescence (Figure 3A and Figure S2). The CCK‐8 and EdU assays showed that the overexpression of LCN2 repressed chondrocyte proliferation (Figure 3B,C). As shown by the flow cytometry assays, the percentage of cells in the G0/G1 phase was markedly higher in the pHBLV‐LCN2 group than in the negative control group (Figure 3D,E). *LCN2*‐overexpressing cells were arrested in the G0/G1 phase.

To assess the role of *LCN2* in hypertrophic features and endochondral ossification of chondrocytes, we measured the levels of type X collagen (*COLX*), *OCN*, *OPN* and *RUNX2* following *LCN2* overexpression. Additionally, von Kossa and alkaline phosphatase (ALP) staining were performed. The osteoblast differentiation marker genes, collagen type X and *RUNX2* showed obvious downregulation according to western blotting and quantitative real‐time polymerase chain reaction (RT‐qPCR) analyses after *LCN2* overexpression (Figure 3F,G), indicating that chondrocyte hypertrophy was inhibited. In addition to *COLX* and *RUNX2*, the levels of the osteogenic genes *OCN* and *OPN* were downregulated following *LCN2* overexpression (Figure 3H,I). ALP activity was reduced, and von Kossa staining revealed a decrease in mineralization (Figure 3J,K). These findings confirm that the overexpression of *LCN2* suppresses chondrocyte proliferation, hypertrophic features and endochondral ossification. Furthermore, an in situ hybridization assay revealed that LCN2 was localized in human chondrocytes and femoral growth plates of SD rats (Figure S5).

### 3.4 *LCN2* overexpression results in short stature in rats by repressing food intake and impairing bone formation in the growth plate

**FIGURE 4:**
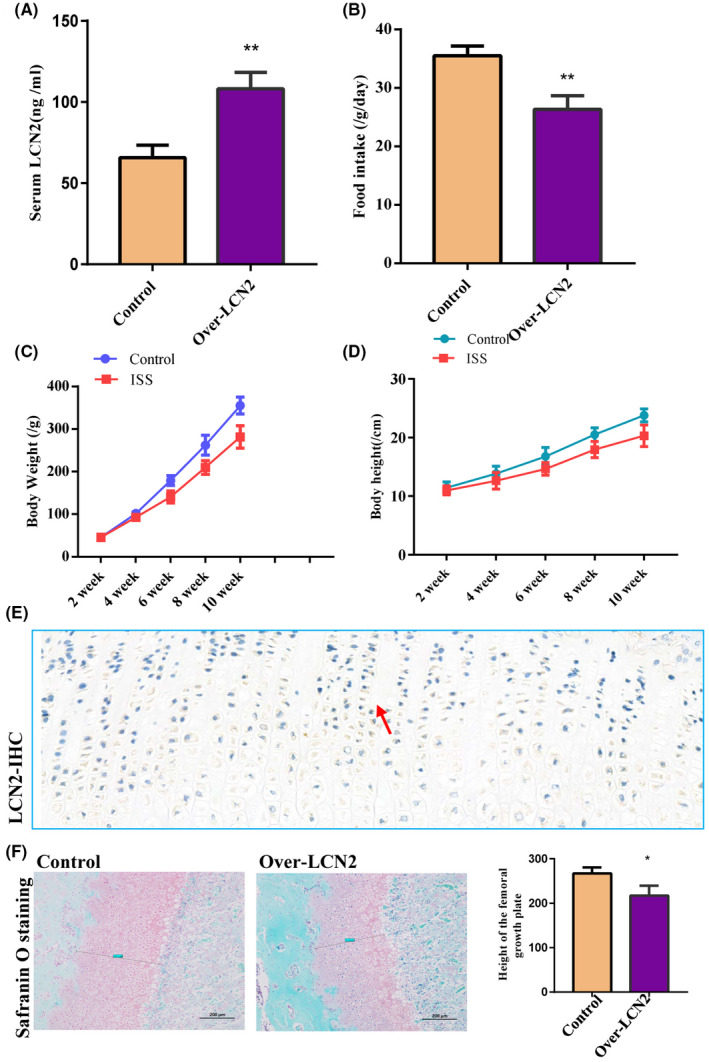
The localization of LCN2 in human chondrocytes and the neonatal femur growth plate of rats was verified, and the body height and weight of rats were decreased after LCN2 overexpression. (A) The serum LCN2 levels were upregulated after daily intraperitoneal administration of LCN2. (B) The average daily food intake was reduced by 25% after the LCN2 overexpression. (C, D) The body weight and body height gain decreased by 15% and 14%, respectively. (E) Immunohistochemistry observed that Lipocalin 2 was dominantly expressed in the proliferative region of the distal femoral growth plate in 8‐week‐old male rats. (F) Safranin O staining revealed that the height of the femoral growth plate in the LCN2‐ overexpressing rats was shorter than that in the control rats. The data are presented as the mean ± SD. *n* = 3. *T*‐test, *p < 0.05, **p < 0.01 vs. control. ALP, alkaline phosphatase; LCN2, Lipocalin 2

To observe whether *LCN2* can result in short stature in vivo, *LCN2* was overexpressed in wild‐type rats via daily intraperitoneal administration (150 ng/g) for 20 or 40 days, according to Mosialou's method.^29^ Serum LCN2 levels were higher in the *LCN2* overexpression group than in the control group (1.74‐fold, Figure 4A). The average daily food intake, body weight and body height gain of the *LCN2* overexpression group declined by 25%, 15% and 14%, respectively, compared to that of the control group (Figure 4B–D).

**FIGURE 5:**
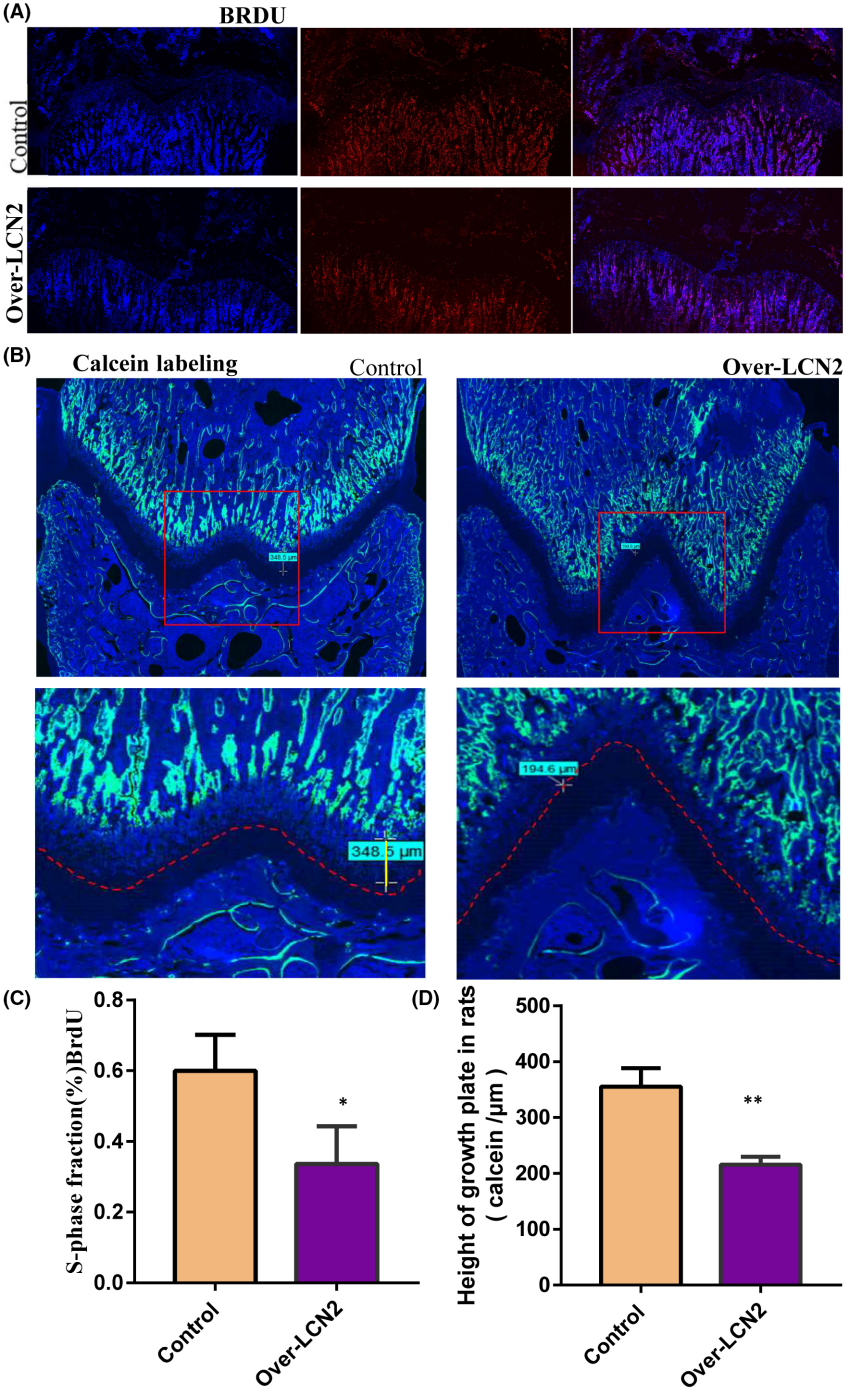
The proliferation and endochondral ossification of the growth plate in the rats were suppressed following LCN2 overexpression. (A) Positive expression of BrdU labeling in the proliferative region of the growth plate in the rats overexpressing LCN2 was lower than that in the control rats. (B) Calcein labeling demonstrated the new bone formation rate in the rats overexpressing LCN2 was obviously decreased compared to that in control rats. (C) Statistical analysis showed that positive expression of BrdU labeling in the proliferative region of the growth plate in the rats overexpressing LCN2 was lower than that in the control rats. (D) Statistical analysis showed that calcein labeling demonstrated the new bone formation rate in the rats overexpressing LCN2 was obviously decreased compared to that in control rats. The data are presented as the mean ± SD. *n* = 3. *T*‐test, *p < 0.05, **p < 0.01 vs. control. LCN2, Lipocalin 2

**FIGURE 6:**
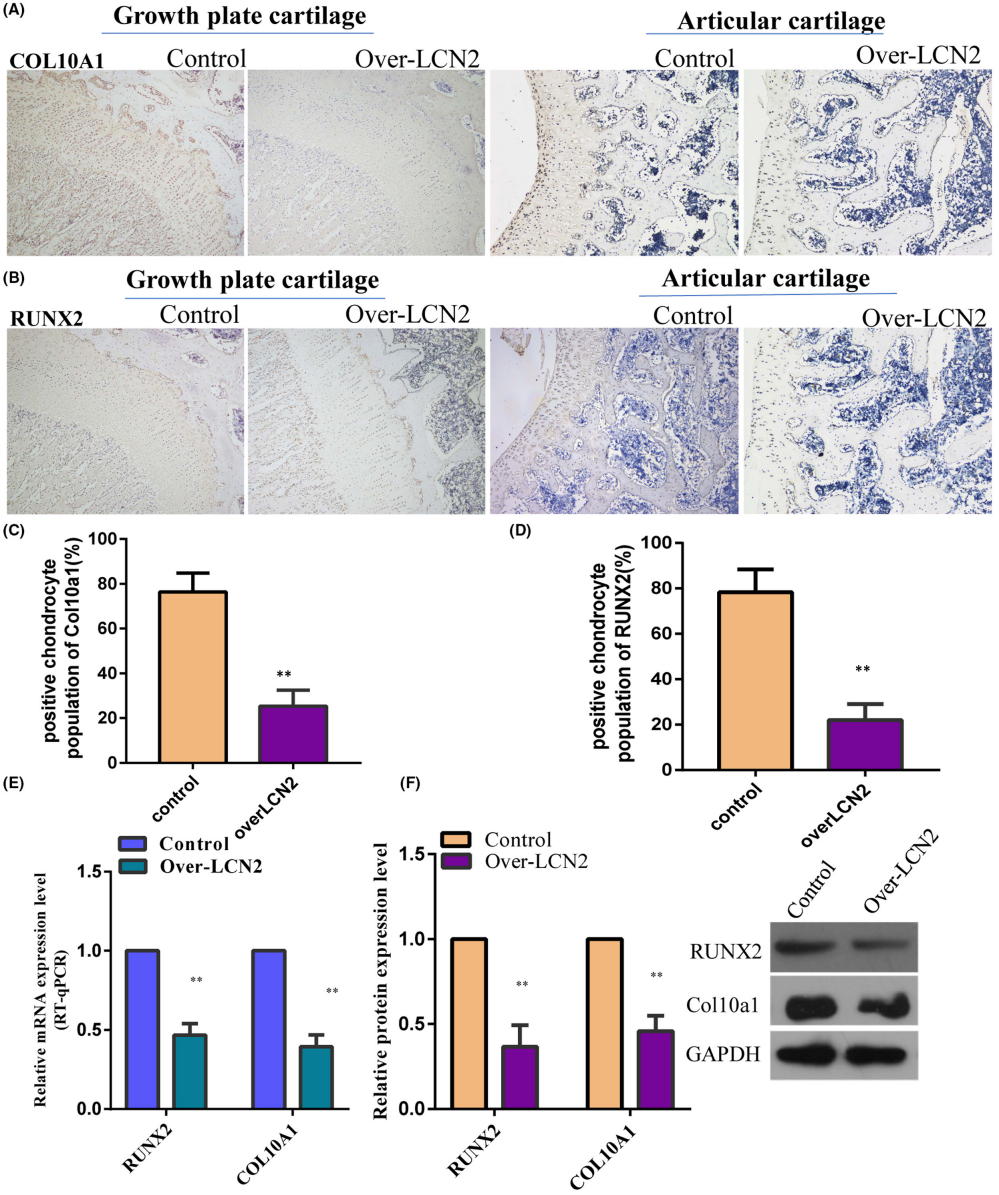
The hypertrophy of growth plate chondrocytes in the rats was suppressed following LCN2 overexpression. (A–D) Immunohistochemistry revealed that the expression of collagen type X and RUNX2 in the growth plates, and articular cartilage was obviously downregulated in the rats overexpressing LCN2. (E, F) The downregulated expression of collagen type X and RUNX2 in the growth plates and articular cartilage of the rats overexpressing LCN2 was further verified via western blot and qPCR. The data are presented as the mean ± SD. *n* = 3. *T*‐test, **p < 0.01 vs. control. LCN2, Lipocalin 2; RUNX2, runt‐related transcription factor 2

**FIGURE 7:**
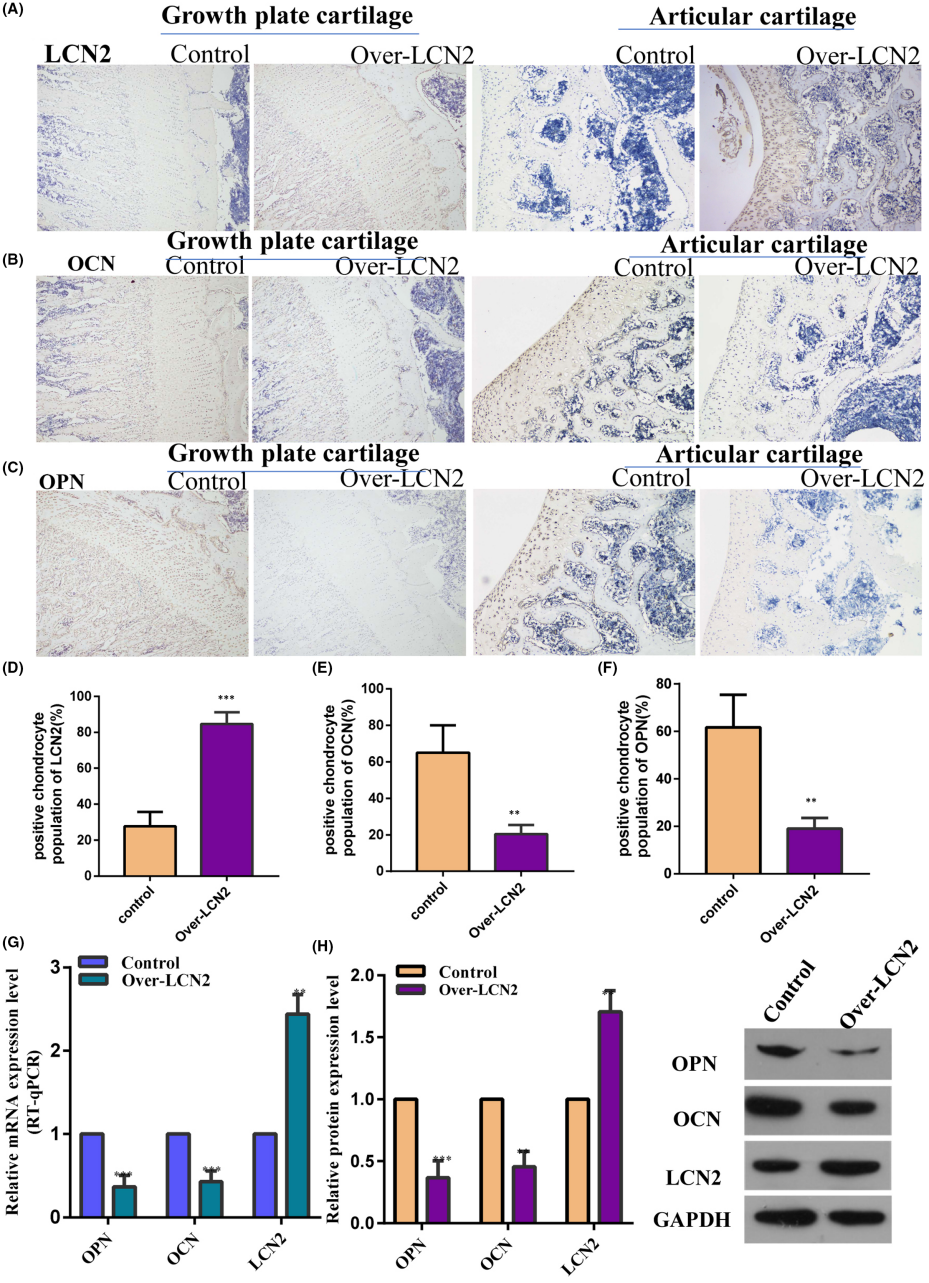
The expression of osteogenic marker genes, such as OCN and OPN, in the growth plate of rats was inhibited following LCN2 overexpression. (A–F) Immunohistochemistry revealed that the expression of OCN and OPN in the growth plates and articular cartilage was obviously downregulated in the rats overexpressing LCN2. (G, H) The downregulated expression of OCN and OPN in the growth plates and articular cartilage of the rats overexpressing LCN2 was further verified via western blot and qPCR. The data are presented as the mean ± SD. *n* = 3. *T*‐test, **p < 0.01, ***p < 0.001 vs. control. LCN2, Lipocalin 2; OPN, osteopontin; OCN, osteocalcin

Immunohistochemistry confirmed that lipocalin 2 was dominantly expressed in the proliferative region of the distal femoral growth plate in 8‐week‐old male rats (Figure 4E). Safranin O staining revealed that the height of the femoral growth plate in *LCN2*‐overexpressing rats was shorter than that in the control rats (Figure 4F). We observed downregulation of BrdU labeling in the proliferative region of the growth plate (Figure 5A,C) and an obvious increase in the new bone formation rate in *LCN2*‐overexpressing rats compared with the control rats (Figure 5B and D). This suggests that *LCN2* overexpression can suppress chondrocyte proliferation and bone growth rate in vivo. Subsequently, the authors measured the expression levels of *COLX* and *RUNX2* in the growth plate. The expression levels of *COLX* and *RUNX2* in femoral growth plates were significantly downregulated in rats overexpressing *LCN2*, compared with the control rats (Figure 6A–F). In addition to *COLX* and *RUNX2*, the expression levels of the osteogenic genes *OCN* and *OPN* were downregulated following *LCN2* overexpression in rats (Figure 7A–H). This indicated that chondrocyte hypertrophy of the growth plate was inhibited in rats after *LCN2* overexpression. Subsequent measurements revealed that the lengths of the femur and tibia in 8‐week‐old rats overexpressing *LCN2* were remarkably decreased compared with the control rats (Figure S6A–D). Taken together, these results provide evidence that upregulated *LCN2* can not only suppress food intake but also impair bone growth in rats. As a result, the rats presented a short‐stature phenotype.

### 3.5 Bone formation of the growth plate is recovered after *LCN2* overexpression is stopped in rats

To address whether the impairment of bone growth caused by *LCN2* overexpression could be reversed in immature rats, we stopped *LCN2* overexpression after 20 days. In this study, the rats were divided into three groups: group 1, group 2 and the control group. In groups 1 and 2, *LCN2* was overexpressed for 20 and 40 days, respectively, via daily intraperitoneal administration. In the control group, 0.9% sodium chloride solution was intraperitoneally administered daily for 40 days. The body weight and height in groups 1 and 2 were obviously suppressed compared with those in the control group 20 days after *LCN2* overexpression (Figure S7A,B). Notably, no significant differences were detected between group 1 and the control group, although the body weight, body height and lengths of the femur and tibia in group 2 were smaller than those in the control group 40 days after *LCN2* overexpression (Figure S7C–F). We found that bone growth impairment was reversible in immature rats after *LCN2* overexpression was stopped (Figure S8A–D).

## 4 DISCUSSION

Our proteomics results showed a high correlation between immunity, inflammation and ISS pathogenesis. Thus, the top 10 inflammation‐related proteins were selected to further verify the validity of ELISA. LCN2 showed the most significant difference. LCN2, also known as neutrophil gelatinase‐associated lipocalin, is an innate immune factor^30^ belonging to the lipocalin superfamily and shares 85% homology with humans and mice.^31^ Chen et al.^23^ observed that LCN2 was overexpressed in patients with lupus nephritis and played a crucial role in the pathogenesis of lupus nephritis. Jia et al. ^24^also found that LCN2 is upregulated in adult‐onset Still's disease, which is an autoinflammatory disease. Because both bacterial and autoimmune inflammation can induce the overexpression of LCN2, we compared *LCN2* expression in children with infectious diseases, such as pneumonia and bronchitis, and children with ISS. Plasma LCN2 expression was significantly higher in children with pneumonia and bronchitis than in children with ISS, suggesting that the increased LCN2 levels in children with ISS cannot be attributed to general bacterial infectious diseases. However, it is interesting to note that although CRP levels in children with ISS were within the normal range of clinical reference values, they were higher than the CRP levels in normal control children. The biological replicates in additional independent samples further confirmed that the LCN2 and CRP levels were higher in ISS children than in normal control children. The protein–protein interaction network revealed a correlation between Lcn2 and CRP levels. Subsequently, positive correlations were identified using statistical analysis. Wang et al.^32^ observed that circulating LCN2 was upregulated and positively correlated with high‐sensitivity CRP levels in obesity‐related metabolic and cardiovascular diseases. Ye et al.^33^ also detected a positive correlation between LCN2 and CRP levels in nonalcoholic fatty liver disease. Huang et al.^34^ found that Lipocalin‐2 is closely correlated with glucose metabolism and chronic low‐grade systemic inflammation in type 2 diabetes. Our previous study verified that serum complements C1QA and C1QB were overexpressed in ISS children.^35^ Trovato et al.^36^ detected IFN‐γ at high serum levels in children with ISS. Hashemi et al.^37^ observed a 33.6% incidence of celiac disease among Iranian children with ISS. Celiac disease without typical gastrointestinal symptoms has also been reported in some patients with short stature.^38^ Accumulating evidence indicates that the relationship between autoimmune diseases and chronic low‐grade systemic inflammation with ISS may be a novel perspective for ISS pathogenesis and should be further elucidated via multicenter corporation in future studies.

Mosialou et al.^29^ found that the overexpression of LCN2 through intraperitoneal injection of exogenous proteins suppressed food intake in mature mice and primates.^39^ Further studies have indicated that LCN2 can cross the blood–brain barrier and combine with the melanocortin 4 receptor of the hypothalamus. Finally, the appetite‐suppressing pathway is activated. As early as 2005, Wudy et al.^40^ reported that children with ISS are poor eaters and have a decreased body mass index. To further reveal the role of LCN2 upregulation in ISS pathogenesis, we upregulated the expression of LCN2 in chondrocyte and rats in the present study. Unlike Mosialou et al.^29^ study, we observed that LCN2 not only suppressed food intake but also inhibited chondrocyte proliferation and bone formation in the growth plate of rats. As a result, the rats presented a short‐stature phenotype. Subsequently, LCN2 overexpression was stopped 20 days after the intraperitoneal injection of exogenous LCN2. It is a meaningful finding that bone growth impairment can be reversed in immature rats after LCN2 overexpression is stopped. This suggests that LCN2 could be considered a potential therapeutic target for children with ISS.

In the present study, an AUC of 0.895 (95% CI: 0.83–0.94) was obtained. LCN2, with the best cutoff point of >87.87 ng/ml, had a specificity of 82.35% and a sensitivity of 91.18% in discriminating children with ISS from normal control children. Meanwhile, LCN2 with cutoff points of >74.36 ng/ml and > 71.55 ng/ml also showed good performance in discriminating children with ISS from those with GH deficiency and precocious puberty with specificities of 89.71% and 92.65% and sensitivities of 89.71% and 91.18%, respectively. Our results indicated that LCN2 could serve as a potent biomarker for the diagnosis of ISS. In agreement with previous studies,^40^, ^41^, ^42^ we observed that the plasma levels of IGF‐I and IGFBP‐3 were lower in patients with ISS than those in healthy individuals. However, IGF‐I and IGFBP‐3 were not used as biomarkers to distinguish patients with ISS from those with GH deficiency because the latter also presented low plasma levels of IGF‐I and IGFBP‐3, compared with normal individuals. Although serum C‐type natriuretic peptide, procollagen type 1 amino‐terminal propeptide, transthyretin and apolipoprotein A‐II^43^, ^44^, ^45^ have been confirmed to be useful markers for predicting response to GH treatment in ISS, they cannot distinguish patients with ISS from those with GH deficiency and precocious puberty.

In conclusion, this study, to our knowledge, is the first to confirm that serum LCN2 expression is upregulated in children with ISS. Overexpression of LCN2 not only suppresses food intake but also impairs chondrocyte proliferation and bone formation in growth plates. Meanwhile, serum LCN2 also presents high sensitivity and specificity in discriminating children with ISS from GH deficiency, precocious puberty and normal control individuals. In addition, we observed that bone growth impairment could be reversed in immature rats after LCN2 overexpression was stopped. The present study indicates that LCN2 is a valid biomarker for ISS diagnosis and may be a potential target for ISS therapy. However, this study has several limitations. Although LCN2 overexpression in children with ISS has been identified, the reason for this remains uncertain. Our study was conducted at a single center. However, from our perspective, international cooperation should occur. Therefore, a prospective, randomized, controlled, multicenter trial should be conducted in the future to support or refute our findings.

## AUTHOR CONTRIBUTIONS

**Xijuan Liu:** Formal analysis (equal); investigation (equal); project administration (equal); resources (equal); software (equal); writing – original draft (equal). **Jian Zhang:** Software (equal). **Jinghong Yuan:** Data curation (equal); software (equal). **Rui Ding:** Software (equal). **Tao Liu:** Software (equal); supervision (equal); visualization (equal); writing – review and editing (equal). **Jingyu Jia:** Formal analysis (equal); funding acquisition (supporting); writing – review and editing (equal).

## CONFLICT OF INTEREST

All authors have no conflicts of interest to disclose.

## INFORMED CONSENT

Informed written consent was obtained from each patient.

## Supporting information


Figure S1

Figure S2

Figure S3

Figure S4

Figure S5

Figure S6

Figure S7

Figure S8

Data S1

Table S1

Table S2

Table S3

Table S4

## Data Availability

All data generated or analyzed during this study have been included in the article and the supplementary information. Meanwhile, the raw data that support the findings of this study are available from the corresponding author upon reasonable request.
