# Supplementary figures and images for: LCN2 is a new diagnostic biomarker and potential therapeutic target in idiopathic short stature
Source: J Cell Mol Med. 2022 May 24;26(12):3568–81. doi: 10.1111/jcmm.17408 (PMC9189333; doi:10.1111/jcmm.17408)

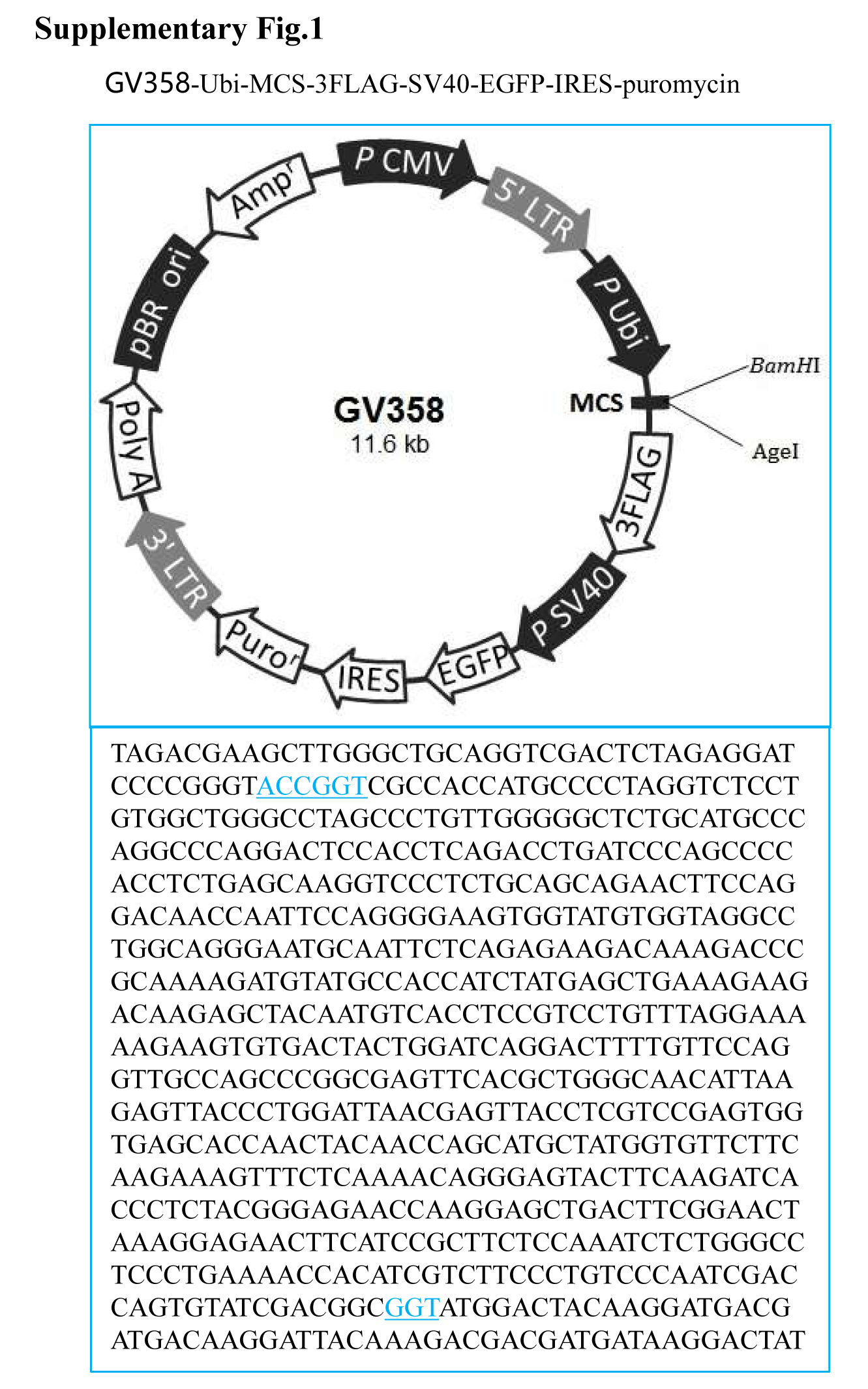

Supplement: Supplementary file 1 — Figure S1 [file JCMM-26-3568-s001.jpg]

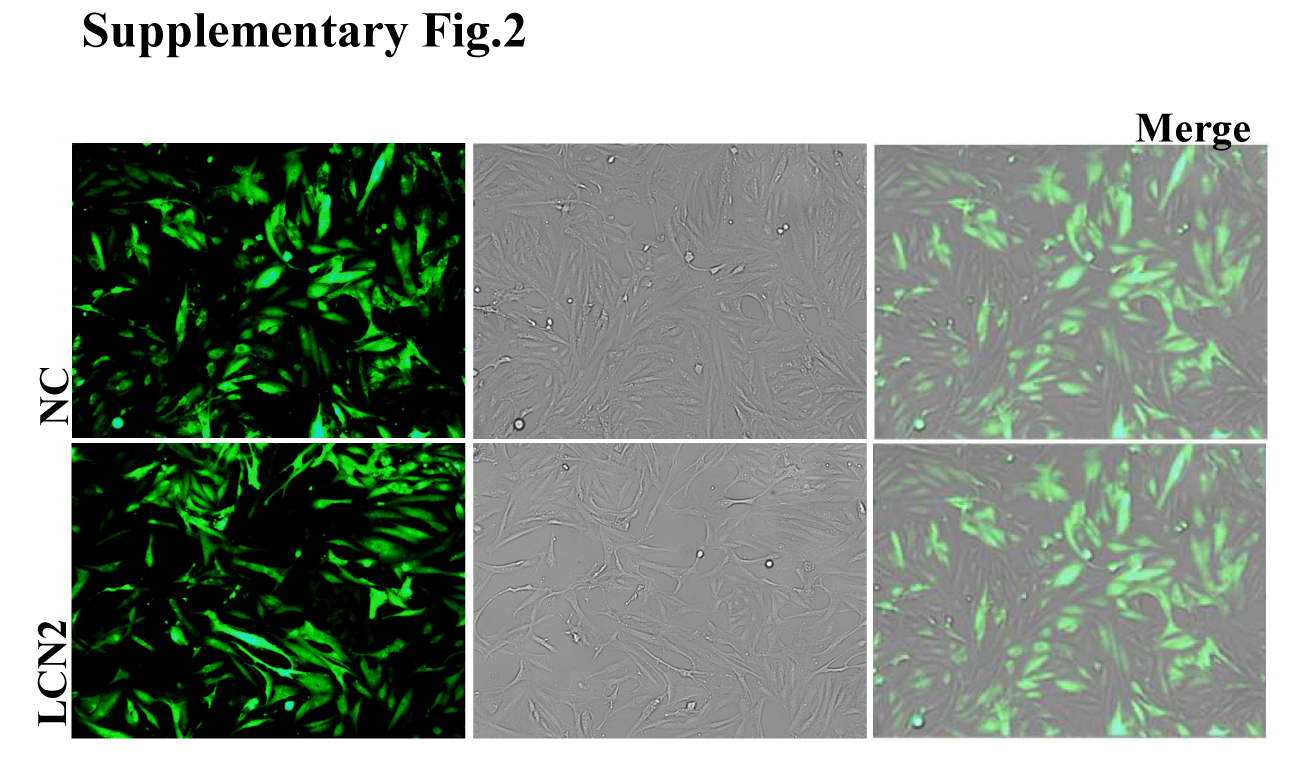

Supplement: Supplementary file 2 — Figure S2 [file JCMM-26-3568-s004.jpg]

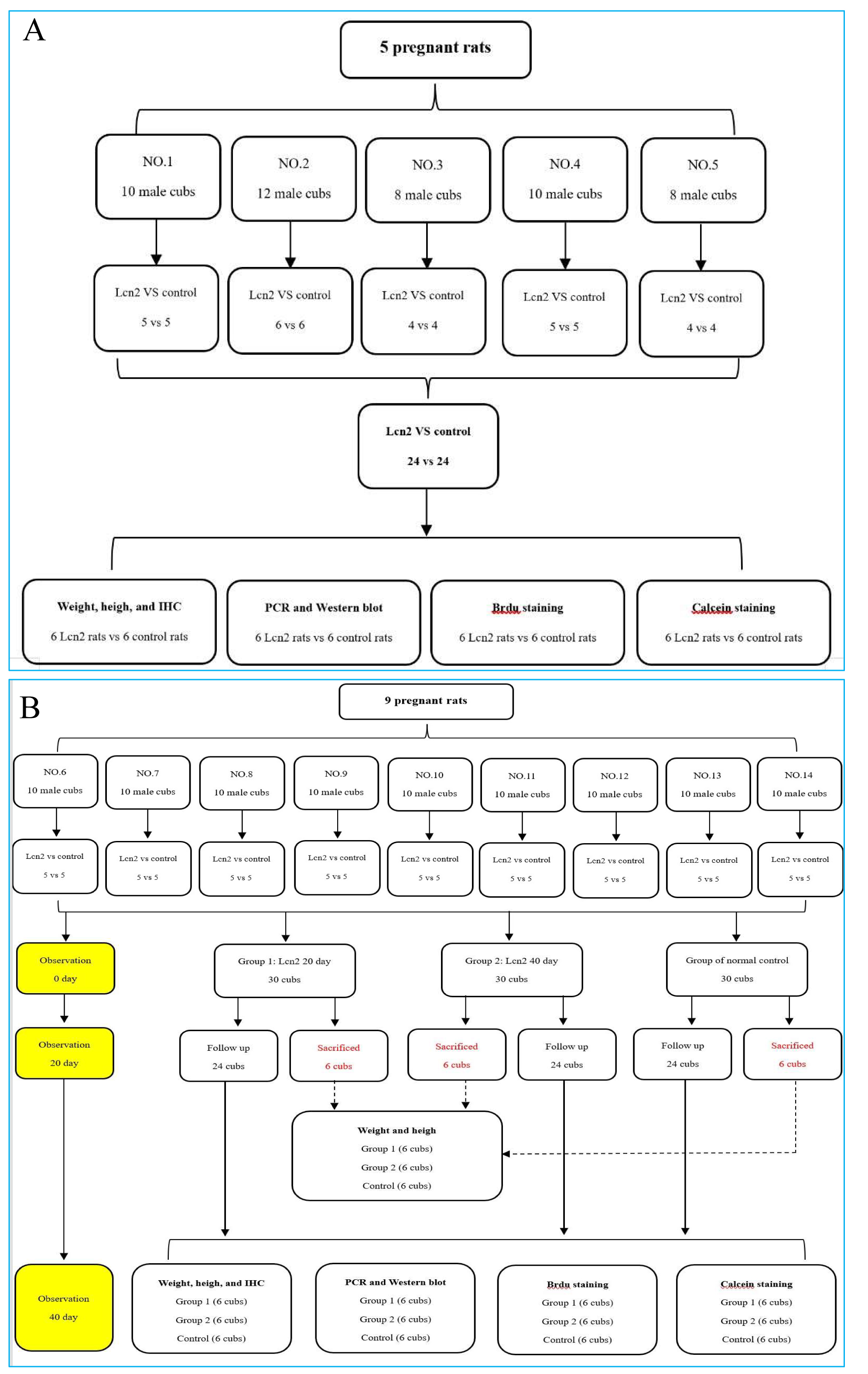

Supplement: Supplementary file 3 — Figure S3 [file JCMM-26-3568-s011.jpg]

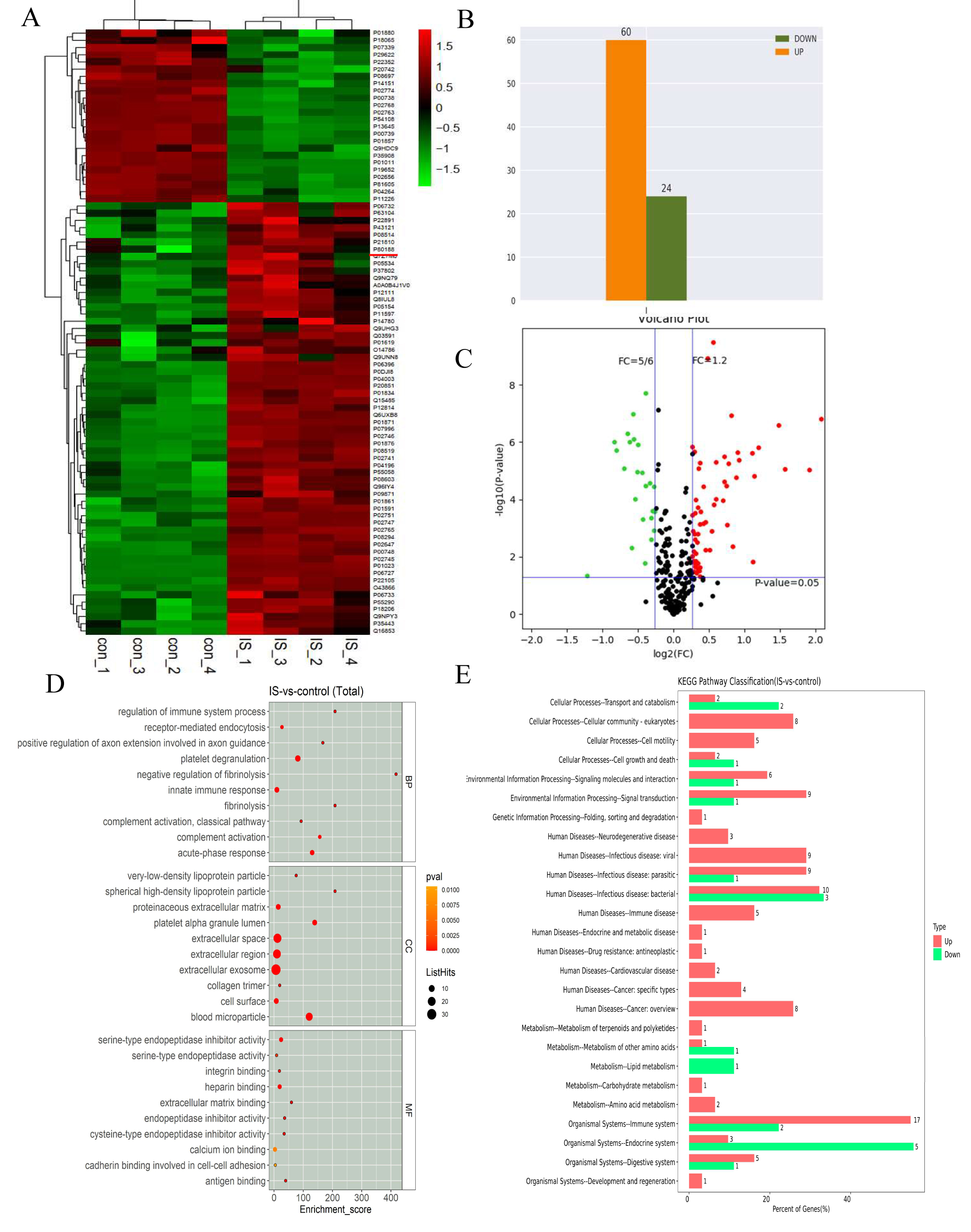

Supplement: Supplementary file 4 — Figure S4 [file JCMM-26-3568-s013.jpg]

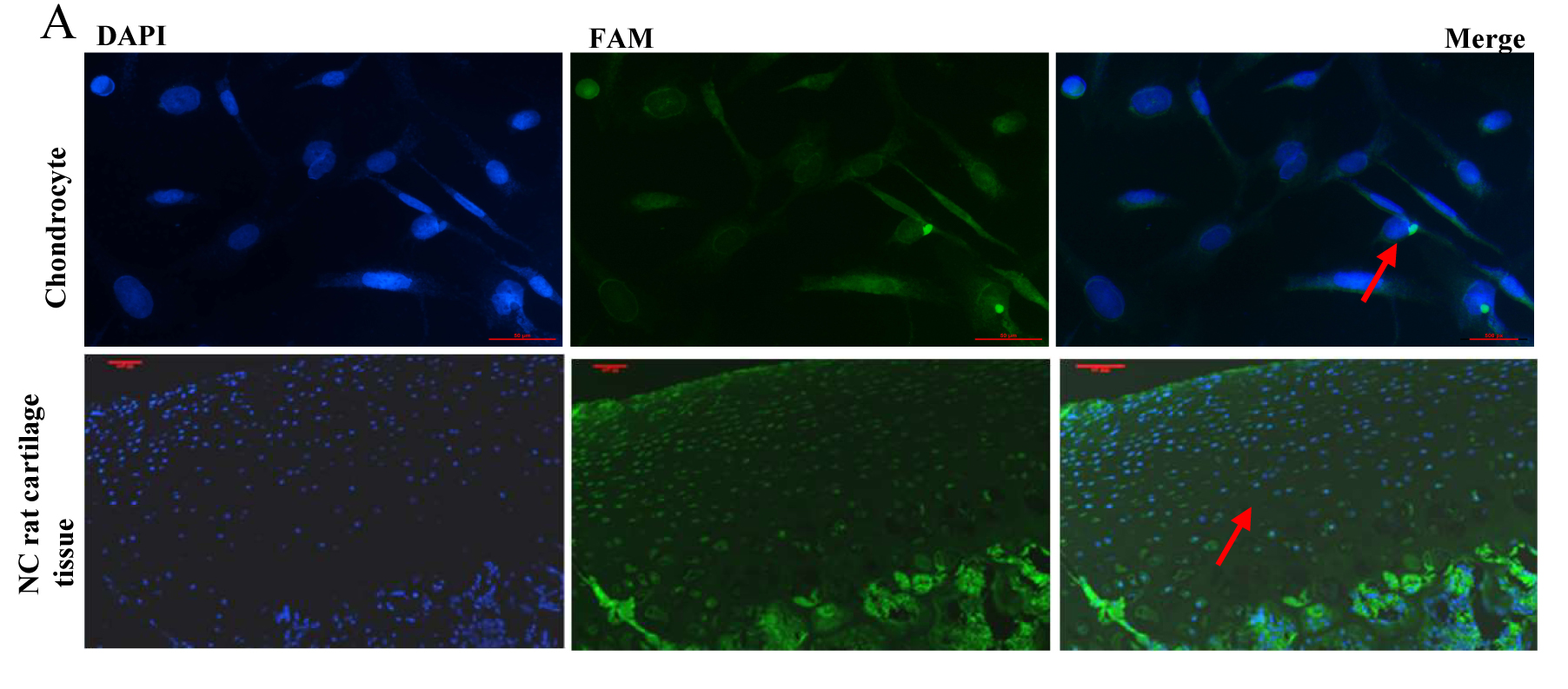

Supplement: Supplementary file 5 — Figure S5 [file JCMM-26-3568-s010.jpg]

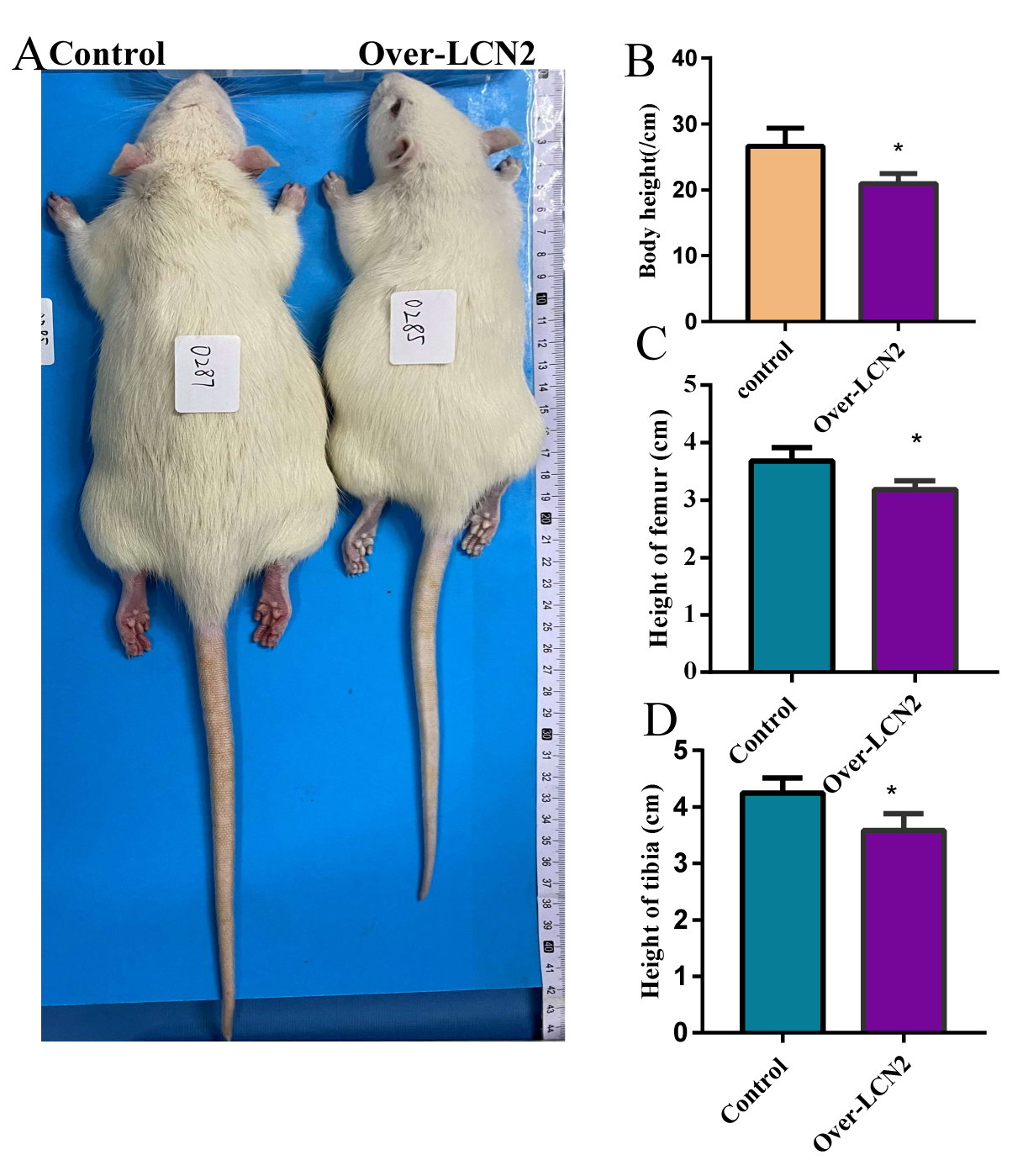

Supplement: Supplementary file 6 — Figure S6 [file JCMM-26-3568-s006.jpg]

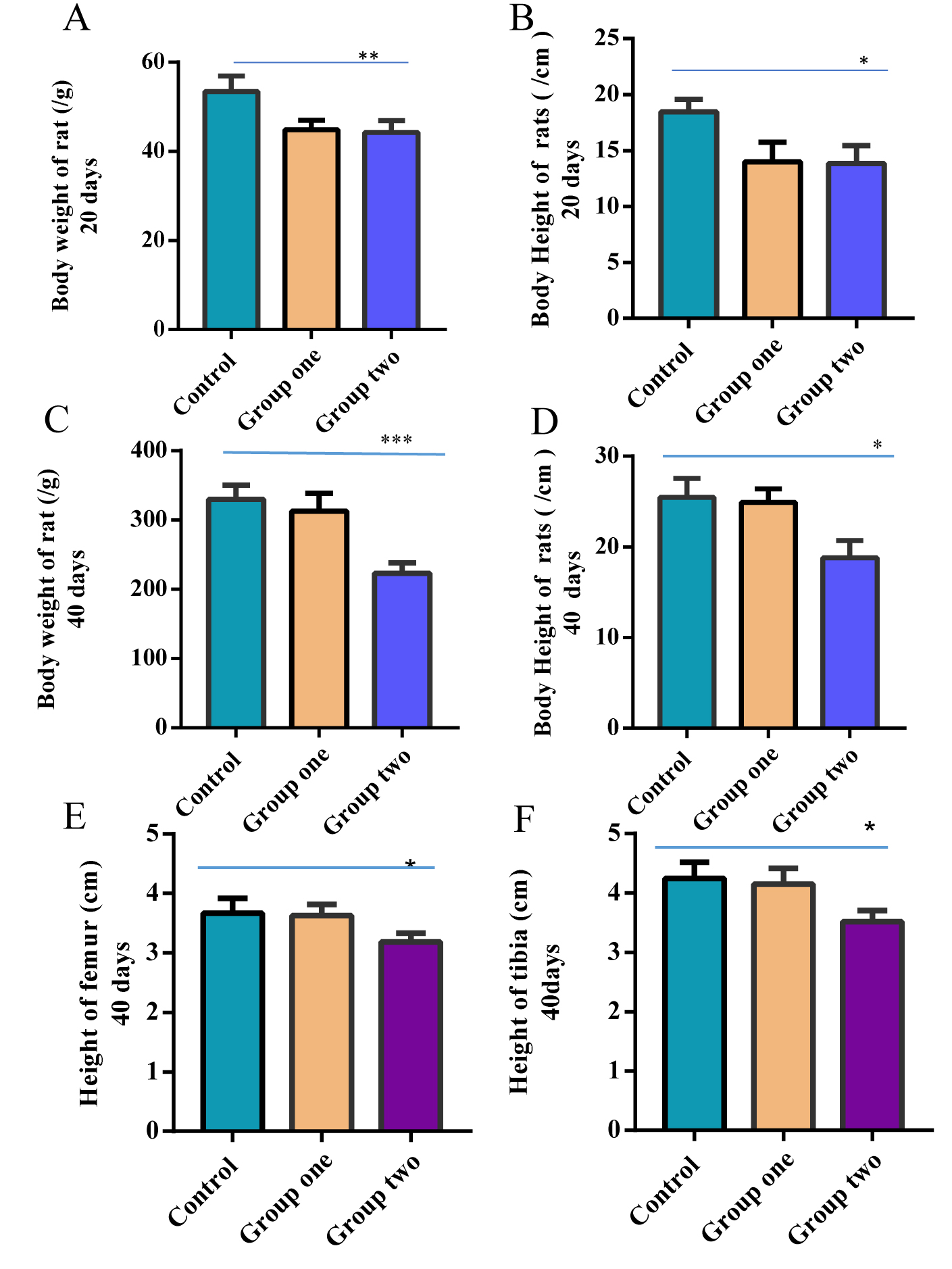

Supplement: Supplementary file 7 — Figure S7 [file JCMM-26-3568-s003.jpg]

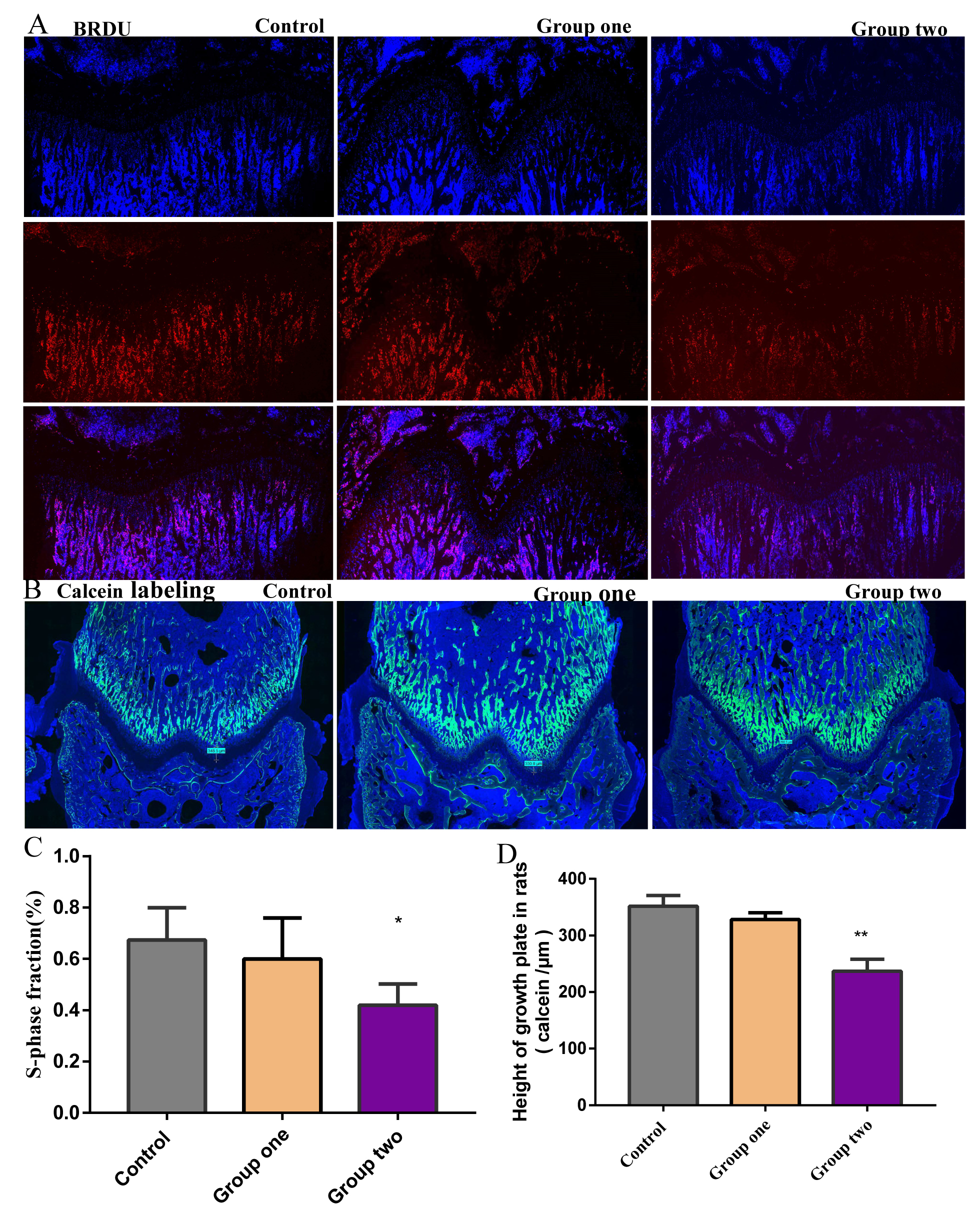

Supplement: Supplementary file 8 — Figure S8 [file JCMM-26-3568-s012.jpg]
