# Supplementary material for: LCN2 is a new diagnostic biomarker and potential therapeutic target in idiopathic short stature
Source: J Cell Mol Med. 2022 May 24;26(12):3568–81. doi: 10.1111/jcmm.17408 (PMC9189333; doi:10.1111/jcmm.17408)
Supplement: Supplementary file 9 — Data S1 [file JCMM-26-3568-s007.docx]

**Supplementary materials and methods**

**Protein preparation and iTRAQ labeling**

Ten microliters of the sample was added directly to the resin slurry in Pierce™ Top 12 Abundant Protein Depletion spin columns (85165, Thermo Scientific Biosciences, Rockford, IL, USA), and the top 12 proteins were removed according to the manufacturer’s instructions. The depleted sample was mixed with in phosphate-buffered saline (PBS 10 mM), NaCl (0.15 M), and 0.02% azide, pH 7.4. The protein content was evaluated using a BCA assay kit (23227, Thermo Scientific Biosciences). Next, 10 μg protein was acquired from each sample and subjected to 12% SDS-PAGE. After separation, the gel was stained with CBB in accordance with Candiano’s protocol, and the stained gel was analyzed at 300 dpi using an ImageScanner (GE Healthcare, USA). Based on filter-aided sample preparation, the proteins were digested with trypsin to generate peptides. The peptides were labeled with the iTRAQ Reagent Kit (4381663, ABSCIEX, Framingham, MA, USA) according to the manufacturer’s instructions.

**D-LC-MS/MS**

Reversed-phase (RP) separation was conducted on an 1100 HPLC System (Agilent) equipped with a Zorbax Extend RP column (150 mm × 2.1 mm, 5 μm; Agilent) at 300 μL/min. Tryptic peptides were detected at 210/280 nm UV wavelength. Specimens were collected for 8–60 min, and the eluent was sequentially withdrawn in centrifugal tubes 1–15 every minute. The samples were recycled until the end of the gradient. The separated peptides were lyophilized prior to MS analysis. A fusion mass spectrometer (Thermo Fisher Scientific) coupled with an EasySpray ion source (Thermo Fisher Scientific) was used for the MS analysis. After loading into a capillary trap column (100 μm × 2 cm, RP-C18, Thermo Fisher Scientific), the samples were separated using a capillary analytical column (75 µm × 15 cm, RPC18, Thermo Fisher Scientific) and an EASY-nLCTM 1200 system (Thermo Fisher Scientific) at 300 nL/min. The linear gradient was optimized as follows: 0–10 min, 5–8% B; 10–45 min, 8–22% B; 45–60 min, 22–45% B; 60–64 min, 45–100% B; 64–70 min, 100% B. Full MS scans were conducted at a mass range and resolution of 350–1600 m/z and 15000, respectively, and the automatic gain control (AGC) target was set to 2e5. Higher-energy collisional dissociation (collision energy = 32) was applied to fragment the 10 most intense peaks in the mass spectrum. The MS/MS spectral analysis was conducted at the resolution, AGC target, injection time, and dynamic exclusion of 15000, 5e4, 100 ms, and 30 s, respectively. Data acquisition and analysis were carried out in position ionization mode using a TripleTOF 5600 MS system (ABSCIEX) fitted with a Nanospray III ion source (ABSCIEX) and Proteome Discover 2.3 (Thermo Scientific Biosciences).

**Histomorphological analysis and in situ hybridization**

Femur and tibia samples were extracted after euthanasia via an intraperitoneal injection of pentobarbital (160 mg/kg). One-third of the distal femur and proximal tibia were resected and stored in 4% formaldehyde solution or liquid nitrogen. The specimens were decalcified with 10% EDTA for 2 months. One specimen was then cut into two equal coronal sections that were subsequently embedded in paraffin. Serial sections of 4 µm thickness were obtained for hematoxylin-eosin and safranin O staining and immunohistochemistry (IHC) analysis. Primary antibodies against LCN2, collagen type-X, OPN, OCN, and RUNX2 (1:200 dilution; Beijing Biosynthesis Biotechnology, Beijing, China) and the secondary antibody goat anti-rabbit IgG (HRP conjugate; 1:200 dilution; Beijing Golden Bridge Biotechnology, Beijing, China) were used for the IHC analysis. An Eclipse Ci microscope (Nikon, Tokyo, Japan) was used to capture the IHC images, and the Image-Pro Plus software (Media Cybernetics, MD, USA) was used to quantify the IHC data. The number of chondrocytes (%) was calculated by averaging the cell counts from five randomly selected fields at a magnification of 40×. For in situ hybridization, human chondrocytes were exposed to 500 ng/mL fluorescein amidite (FAM)- and Cy3-labeled probes (Mus LCN2 5′-FAM-CTGGTCCTGGTCCCTGACCAGGATGGAGGT-3′; Homo LCN2 5′-FAM-CTGG GCATGCAGAGCCCCCAACAGGGCTAG-3′) for 48 h, and the distribution of LCN2 expression was assessed using a miRCURY LNA miRNA ISH kit (Thermo Fisher Scientific Inc.). Samples of ten-week-old male Sprague-Dawley rats were extracted after euthanasia (160 mg/kg pentobarbital, intraperitoneal injection) and then stored in 4% formaldehyde solution. After decalcification in EDTA solution (10%) for one month, the specimens were embedded in paraffin, sectioned at 4-µm thickness, and subjected to in situ hybridization.

**BrdU and calcein staining**

The proliferation rate of femur growth plates was calculated using BrdU staining. BrdU (Sigma-Aldrich, St. Louis, MO, USA) was injected intraperitoneally (150 ng/g) 2 h before euthanizing the rats. Subsequently, one-third of the distal femur was dissected, paraffin-embedded, and sectioned to a thickness of 4 µm. The BrdU IHC staining was performed using a BrdU In Situ Detection Kit (BD Biosciences, CA, USA), followed by methyl green counterstaining. The BrdU-positive cells in the femur growth plate were counted and compared between the LCN2 expression and normal control groups using Lui’s method. Bone growth rate was determined using calcein staining. Calcein (Sigma-Aldrich, MO, USA) was injected intraperitoneally (150 ng/g) 7 days before the rats were euthanized. One-third of the distal femur was dissected and embedded in paraffin. After rehydration and counterstaining with 4′,6-diamidino-2-phenylindole (DAPI), sections of the distal femur were mounted with Fluoromount G (Thermo Fisher Scientific Inc.). Considering that calcein has a short half-life in the circulatory system, the formation of bone matrix will only be labeled 48 h after calcein injection. After fluorescence microscopic analysis (Keyence BZ-X700; Keyence Corp., Osaka, Japan), the longitudinal bone growth was calculated by measuring the distance between the respective green fluorescent bands (casein label) and the metaphyseal chondro-osseous junction. For instance, the bone growth rate (μm/d) in 7-day-old mice after calcein injection on day 6 and euthanasia on day 8 was measured by dividing the distance between the green fluorescent signals and the chondro-osseous junction by 2.

**Alkaline phosphatase and Von Kossa staining**

von Kossa staining was performed to assess the in vitro formation of mineralized nodules in chondrocytes. After rinsing twice with PBS, the chondrocytes (72 h after transfection of the overLCN2) were fixed in a 95% EtOH for 10 min at room temperature (RT; 22 ± 3 °C). Subsequently, the chondrocytes were stained with von Kossa silver solution for 1 min and exposed to UV light at RT for 10 min. After rinsing with dH_2_O, the chondrocytes were incubated with hypo solution (1 mL) at RT for 1 min, stained with hematoxylin solution (1 mL) at RT for 2 min, and stained with eosin at RT for 1 min. The presence of mineralized nodules was indicated by a dark brown or black color. For alkaline phosphatase staining, the chondrocyte cell lysates were rinsed three times in PBS, fixed with 4% paraformaldehyde at RT for 15 min, and incubated with BCIP/NBT Alkaline Phosphatase Color Development Kit (Solarbio Inc., Beijing, China) for 48 h in the dark.

**RT-qPCR and Immunoblotting**

An Eastep Super RNA Extraction Kit (Promega Biotech Co., Ltd., Beijing, China) was used to extract total RNA from each frozen specimen. Reverse transcription was performed using the PrimeScript™ RT Reagent Kit with gDNA Eraser (TaKaRa, Japan). The mRNA levels of *LCN2*, *COLX*, *OCN*, *OPN*, and *RUNX2* were quantified using an ABI Q6 PCR system (Applied Biosystems, Foster City, CA, USA) using the primers shown in Supplementary Table 1. The RT-qPCR cycle (TB Green® Premix Ex Taq™ II, TaKaRa, Japan) was as follows: 95 °C for 10 min, followed by 40 cycles of 10 s at 95 °C, and 34 s at 60 °C. GAPDH was used as an internal control. Each reaction was conducted in triplicate using three independent cell preparations. After normalization, the 2-ΔΔCt method was used to calculate the relative mRNA expression level of each gene. Possible errors caused by variations in RNA amount and transcription efficiency were eliminated using an internal control.

**Immunoblotting**

Total protein was isolated from the chondrocytes of the two groups via cell lysis (Total Protein Extraction Kit, Applygen Technologies, Inc., Beijing, China) and then measured using a BCA assay (Thermo Fisher Scientific). After separation by SDS-PAGE (Beijing Biosynthesis Biotechnology, Beijing, China), the proteins were transferred onto polyvinylidene difluoride (PVDF) membranes. Subsequently, the membrane was blocked with skim milk (Solarbio, Inc.) for 1 h and incubated overnight with anti-LCN2, anti-COLX, anti-OPN, anti-OCN, and anti-RUNX2 antibodies at 4 °C. The primary antibodies were obtained from Abcam (Cambridge, MA, USA) at a dilution of 1:2000. After rinsing with 1 × TBST (Solarbio, Inc.), the membrane was incubated with HRP-conjugated rabbit anti-mouse (1:3000 dilution; Abcam, USA) secondary antibody at RT for 1 h. Finally, the immunoblots were detected with chemiluminescent horseradish peroxidase substrates (Pierce, Rockford, IL, USA). GAPDH (1:3000, Abcam, USA) was used as the internal control. In addition, we detected multiple proteins on the same immunoblot membrane using the WB stripping buffer (NO: 21059; Thermo Fisher Scientific) and by reprobing the membrane with different primary antibodies. The changes in gray values were determined using the Image Lab V5.2.1 software.
