# Supplementary material for: LCN2 is a new diagnostic biomarker and potential therapeutic target in idiopathic short stature
Source: J Cell Mol Med. 2022 May 24;26(12):3568–81. doi: 10.1111/jcmm.17408 (PMC9189333; doi:10.1111/jcmm.17408)
Supplement: Supplementary file 10 — Table S1 [file JCMM-26-3568-s002.doc]

Supplementary Table 1

Primers used for qRT-PCR analysis of mRNA levels.

Target ID Primer sequence 5’-3’

LCN2 F: AAGGCAGCTTTACGATGTACAGC

R: CTTGCACATTTAGCTGTGTACC

RUNX2 F: ACTTCCTGTGCTCCGTGCTG

R: TCGTTGAACCTGGCTACTTGG

COL10 F: GCAGCATTACGACCCAAGAT

R: CATGATTGAACTCCCTGAAG

OPN F: CCAGCCAAGGACCAACTACA

R: AGTGTTTGCTGTAATGCGCC

OCN F: GCACCACCGTTTAGGGCAT

R: CGTTCCTCATCTGGACTTTATTTTG

GAPDH F: GGAGCGAGATCCCTCCAAAAT

R: GGCTGTTGTCATACTTCTCATGG
